# Evaluation of a recombination-resistant coronavirus as a broadly applicable, rapidly implementable vaccine platform

**DOI:** 10.1038/s42003-018-0175-7

**Published:** 2018-10-29

**Authors:** Rachel L. Graham, Damon J. Deming, Meagan E. Deming, Boyd L. Yount, Ralph S. Baric

**Affiliations:** 1 Department of Epidemiology, The University of North Carolina at Chapel Hill, 2107 McGavran-Greenberg, CB 7435, Chapel Hill, NC 27599 USA; 2 Department of Microbiology and Immunology, The University of North Carolina at Chapel Hill, Chapel Hill, NC 27599 USA; 3 Present Address: Food and Drug Administration, 10933 New Hampshire Avenue, Bldg 22, Rm 6170, Silver Spring, MD 20993 USA; 4 Present Address: University of Maryland Medical Center, Department of Medicine, Division of Infectious Disease, Institute of Human Virology, 725 West Lombard Street, Room 211A, Baltimore, MD 21201 USA

## Abstract

Emerging and re-emerging zoonotic viral diseases are major threats to global health, economic stability, and national security. Vaccines are key for reducing coronaviral disease burden; however, the utility of live-attenuated vaccines is limited by risks of reversion or repair. Because of their history of emergence events due to their prevalence in zoonotic pools, designing live-attenuated coronavirus vaccines that can be rapidly and broadly implemented is essential for outbreak preparedness. Here, we show that coronaviruses with completely rewired transcription regulatory networks (TRNs) are effective vaccines against SARS-CoV. The TRN-rewired viruses are attenuated and protect against lethal SARS-CoV challenge. While a 3-nt rewired TRN reverts via second-site mutation upon serial passage, a 7-nt rewired TRN is more stable, suggesting that a more extensively rewired TRN might be essential for avoiding growth selection. In summary, rewiring the TRN is a feasible strategy for limiting reversion in an effective live-attenuated coronavirus vaccine candidate that is potentially portable across the Nidovirales order.

## Abstract (web summary)

Rachel Graham et al. show that coronaviruses with extensively rewired transcription regulatory networks are effective vaccine candidates against Severe Acute Respiratory Syndrome Coronavirus. The reversion of live-attenuated vaccine strains to virulence can be avoided with these vaccines.

## Introduction

Emerging and re-emerging zoonotic viral diseases are major threats to global human health, economic stability, and national security^1–6^. The incidence of human zoonotic disease is estimated to surpass 1 billion cases per year, with novel emerging infectious diseases accruing hundreds of billions of dollars in economic losses^7,8^, losses that are greatly magnified when new emerging viruses such as coronaviruses (CoVs) devastate economically critical livestock populations across the globe. With the continued encroachment of human populations into animal habitats and our close contact with domesticated animals, zoonoses will continue to increase as the human and livestock population numbers and density expand over the next century. In fact, a recent study recognized that the majority of emerging infectious disease events have origins in wildlife^3,8^, underscoring the importance of developing broadly applicable strategies for vaccine design for virus families that are harbored within extensive zoonotic pools.

Vaccines are well established in their capacity to reduce viral disease burden. Live-attenuated vaccines, because they can elicit balanced innate and adaptive—and often lifelong—protective immune responses, including lactogenic immunity, are ideal candidates for vaccine development in humans and animals^1^. However, their utility as broadly applicable vaccine platforms has long been limited by risks of reversion of attenuated vaccine strains to virulence, largely because the stability of the attenuation cannot be clearly evaluated or assured.

**Fig. 1:**
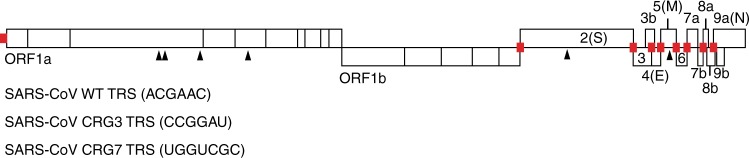
Schematic of the rewired TRN SARS-CoV mutants. The SARS-CoV genome is depicted, with open reading frames (ORFs) indicated. The locations of mouse-adapted mutations are denoted by black triangles. The location of each characterized TRS is denoted by a red box. The specific TRS core sequences are listed underneath the genome

The emergence of Severe Acute Respiratory Syndrome Coronavirus (SARS-CoV) and Middle-East Respiratory Syndrome Coronavirus (MERS-CoV) in the 21st century emphasizes the threat of pandemic viral infections originating from cross-species transmission events^9–11^. These highly pathogenic variants are prime models for the development of broad-based strategies for evaluating live virus vaccines for the Nidovirales order. Coronaviruses (CoVs) all reproduce with conserved replication strategies, emphasizing the strength and rapidly adaptable potential of a vaccine design platform that takes advantage of this biology. CoVs replicate and transcribe subgenomic RNAs (sgRNAs) via a discontinuous transcription mechanism mediated by transcription regulatory sequences (TRSs), a series of conserved nucleotide sequences positioned near the 5′-end of the genome and at several locations immediately 5′ of each downstream open reading frame (ORF) (Fig. 1). These TRSs regulate a transcription attenuation program via base-pairing interactions between the leader TRS and body TRSs that results in the production of sgRNAs, from which downstream ORFs are translated. Within the TRS, a 6- to 8-nt core sequence (ACGAAC for SARS-CoV) guides base-pairing and duplex formation between nascent RNA and the leader TRS^12–14^. While this collective TRS arrangement, the transcription regulatory network (TRN), is conserved, our laboratory has shown that it is possible to rewire the guide sequence of the SARS-CoV TRN and produce infectious virus^13^. We also showed that recombination between this rewired TRN virus and wild-type (WT) SARS-CoV was not viable, indicating that recombination-mediated reversion of a CoV vaccine platform featuring a rewired TRN is highly unlikely.

Based on conservation of the TRN biology across CoVs, this report further explores the feasibility of the development of a stably attenuated vaccine platform featuring a completely rewired TRN as a candidate strategy for a broadly applicable, rapidly implementable CoV vaccine platform that is highly resistant to recombination repair and stably attenuated in both young and highly vulnerable mouse models of human disease.

## Results

### The 3-nt TRN mutant is attenuated for virulence

**Fig. 2:**
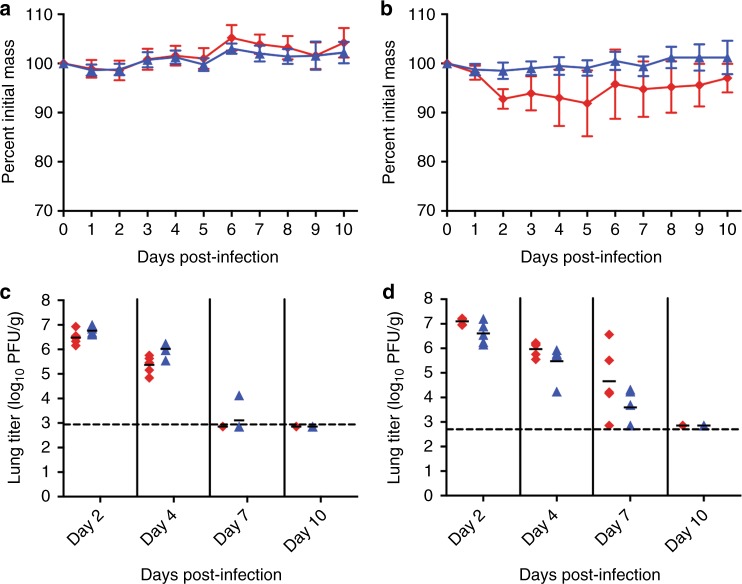
In vivo characterization of the SARS-CoV CRG3 mutant. **a**, **b** Weight loss is depicted as percent initial mass in (**a**) young and (**b**) aged mice. **c**, **d** Viral replication in the lungs is depicted as titer (log_10_ PFU/g) in (**c**) young and (**d**) aged BALB/c mice. Viruses: WT, red diamonds; CRG3, blue triangles. Dashed lines: limits of detection. Error bars depict standard deviation

In a previous study in our laboratory, we demonstrated that the SARS-CoV TRN could be reprogrammed, provided the individual TRSs were replaced with matching sequences^13^. The rewired TRN replaced the conserved 6-nt TRS with a 6-nt cassette that is not used in any other characterized CoVs, encoding a net change of 3 nts (ACGAAC to CCGGAU). Our previous work showed that this rewired TRN was refractory to recombination with WT genomes^13^. Therefore, we tested its replication and pathogenesis in young and aged BALB/c mice. Consistent with earlier reports, WT SARS-CoV only caused weight loss in aged animals. In contrast, CRG3 replicated but caused no weight loss in young (10-week-old) mice (Fig. 2a) and minimal weight loss in aged (12-month-old) BALB/c mice (Fig. 2b) (young mice: *P* *=* 0.25 for titer, Wilcoxon test, *P* *=* 0.37 for weight loss, Mann-Whitney test; old mice: *P* *=* 0.25 for titer, *P* *<* 0.001 for weight loss). Both WT and recombinant viruses replicated to high titers that were detectable on days 2 and 4 post-infection (p.i.) in both young and aged animals, with titers beginning to clear by day 7 p.i. (Fig. 2c, d), as is usually observed in mice infected with SARS-CoV^1^.

### Vaccination with CRG3 protects mice against challenge

**Fig. 3:**
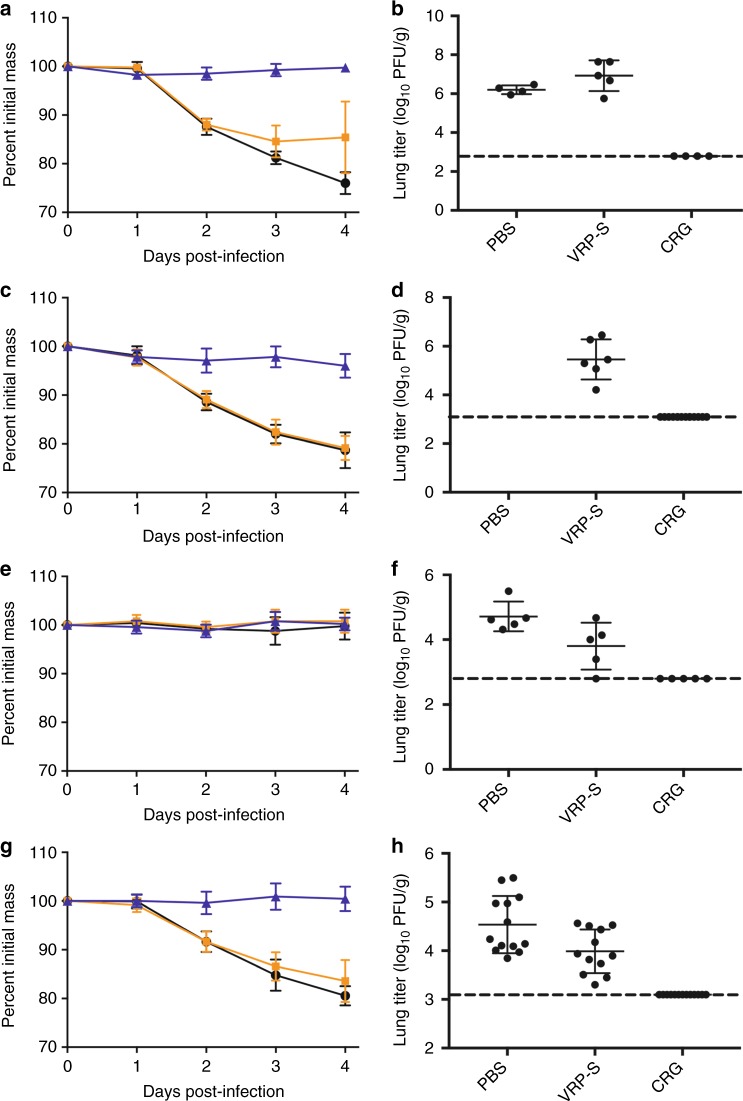
Vaccination of young and aged mice with SARS-CoV CRG3. (**a**, **c**, **e**, **g**) Weight loss upon homologous (**a**, **c**) and heterologous (**e**, **g**) challenge is depicted as percent initial mass in young (**a**, **e**) and aged (**c**, **g**) vaccinated BALB/c mice. **b**, **d**, **f**, **h** Viral replication in the lungs upon homologous (**b**, **d**) and heterologous (**f**, **h**) challenge is depicted as titer (log_10_ PFU/g) in (**b**, **f**) young and (**d**, **h**) aged vaccinated BALB/c mice. Vaccinations: black circles, PBS vaccination; orange squares, VRP-S vaccination; blue triangles, CRG3 vaccination. Dashed lines: limits of detection. Error bars depict standard deviation

The high replication titer and low or absent virulence in young and aged mouse models paired with the inherently recombination-refractory genome suggested that the CRG3 virus would be an ideal vaccine candidate. To test CRG3’s efficacy in protecting against homologous and heterologous challenge, the virus was administered to mice in a single-dose vaccination, alongside viral replicon particles (VRPs) expressing the viral Spike attachment protein (VRP-S) as a control^15^. On day 22 post-vaccination, mice were then challenged with either mouse-adapted SARS-CoV (MA15—homologous challenge) or SARS-CoV expressing the Spike gene from the Himalayan palm civet (*Paguma larvata*) strain HCSZ6103 (heterologous challenge). Mice were then observed for morbidity and mortality, and surviving animals were euthanized on day 4 p.i. Upon homologous challenge, CRG vaccination was protective against weight loss and mortality in young and aged mice, with no detectable viral titer in the lungs at 4 days p.i., in contrast to PBS and VRP-S subcutaneous vaccination (Fig. 3a–d, Supplementary Fig. 1A-B). Importantly, against heterologous challenge, while both CRG3 and VRP-S vaccines were protective in young mice, only CRG3 was protective in aged mice against replication and mortality, with no detectable titer in the lungs at 4 days p.i. (Fig. 3e–h, Supplementary Fig. 1C–D). CRG vaccination induced SARS-CoV neutralizing antibody titers approaching 4 log_10_ in both young and aged mice, nearly 2-fold higher than the titers induced by VRP-S vaccination (*P* < 0.0001, two-way ANOVA) (Supplementary Fig. 1E). Moreover, CRG vaccination largely protected aged mice from the extensive inflammatory cell infiltration, tissue damage, perivascular cuffing, edema, and septal thickening observed in PBS- and VRP-S-vaccinated mice (Supplementary Fig. 2).

### CRG3 reverts with pandemic-associated mutations

**Fig. 4:**
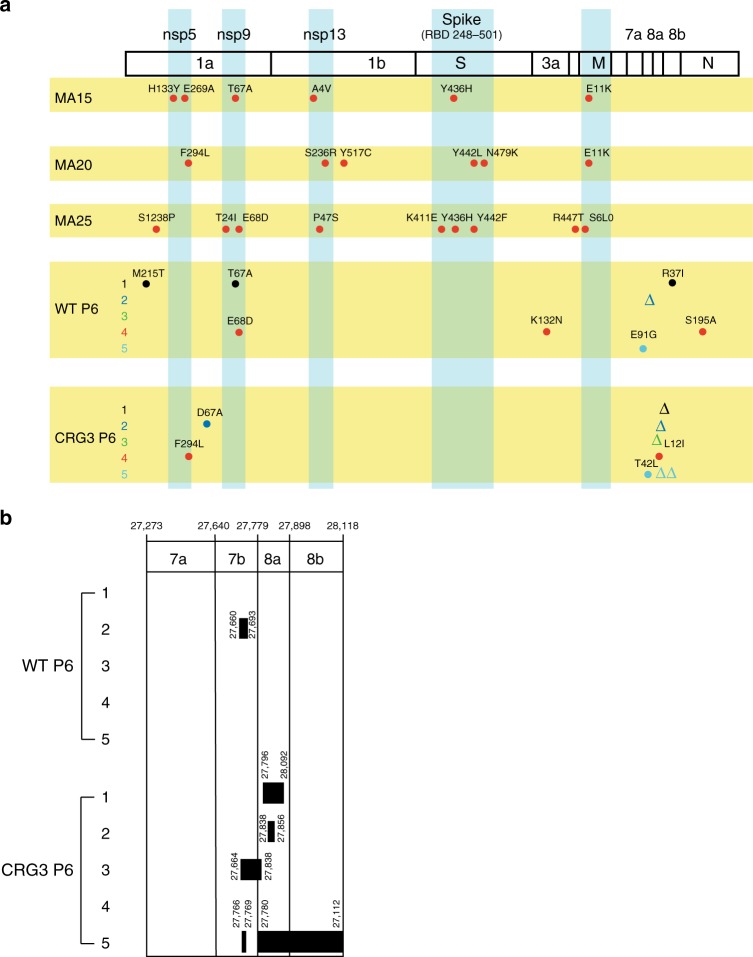
Accumulation of second-site mutations upon passage of SARS-CoV CRG3. **a** The SARS-CoV genome is depicted at the top. The mutations identified in the 3 published mouse-adapted SARS-CoV genomes (MA15, MA20, and MA25) are shown in the top 3 yellow sections immediately below the genome. The mutations identified via Sanger sequencing in 5 individual plaque isolates of WT SARS-CoV and CRG3 after 6 passages (WT P6 and CRG3 P6, respectively) are depicted in the bottom 2 yellow sections. Nonsynonymous mutations that led to amino acid substitutions are identified by their WT amino acid residue, the residue number in the mature protein, and the substituted residue. **b** The genome bounds of the SARS-CoV ORF 7a, 7b, 8a, and 8b accessory proteins are denoted. Individual rows indicate each of 5 plaque isolates from WT SARS-CoV after 6 passages (WT P6) and CRG3 after 6 passages (CRG3 P6). Deletions are indicated with black boxes in each row, with the bounds of the deletions specified

A live-attenuated vaccine candidate should demonstrate phenotypic stability in infected host populations. Therefore, to test its resistance to reversion to virulence, CRG3 was subjected to five independent serial passages in parallel with WT SARS-CoV in aged (14-month-old) BALB/c mice. Over the course of six 4-day passages, all CRG3 passaged viruses acquired a virulent phenotype, with weight losses and percent survival curves mirroring the kinetic rates of the emergence of virulence seen with WT passaged viruses (Supplementary Fig. 3). To attempt to identify the genotypic causes of this phenotypic reversion to virulence, the genomes of 5 independent plaque isolates of the passage 6 viruses (WT P6 and CRG3 P6) were submitted to Sanger sequencing. Surprisingly, and contrary to expectations, no mutations in either the Spike or the Membrane protein—both of which were targets for mutational selection in 3 separate adaptations of SARS-CoV Urbani to mice (Fig. 4a)—were identified in WT or CRG3 revertants. Instead, 4 of 5 CRG3 P6 isolates showed evidence of often large deletions in the accessory ORFs 7b, 8a, and 8b (Fig. 4a, b). These deletions, often in-frame, were reminiscent of several incidences of host range-associated deletions identified in human isolates from the 2003 SARS-CoV epidemic, including a 29-nt deletion in ORF8 relative to the Himalayan palm civet strain^16^ and 82-nt^17^ and 386-nt^18^ deletions in this same region. In contrast, deletions in accessory ORFs were rare in virulent WT revertants. Rather, scattered nonsynonymous mutations were identified in ORF1a, ORF3a, ORF8a, and ORF9. Notably, only 1 WT revertant exhibited a small deletion in ORF7b. Collectively, these findings demonstrated that this region of the SARS-CoV genome is inherently unstable when subjected to replication pressure, especially in aged animals, and indicated that a TRN-rewired virus would require additional stabilizing mutations to be feasible as a vaccine candidate.

### An attenuated SARS-CoV mutant with a 7-nt TRN replacement

The demonstration of second-site reversion to virulence upon passage of CRG3 suggested that a more ideal TRN-rewired vaccine candidate would be one that would be less likely to phenotypically revert in vivo. CoV TRS networks are finely tuned and regulate the expression of both highly abundant and low-frequency mRNA transcripts; thus, small changes in the TRS might subtly alter the regulation of subgenomic transcripts^13^. Moreover, the exact regulatory milieu around each TRS is uncertain beyond the recognition that both, up- and downstream sequences can influence mRNA expression efficiency, perhaps in a TRN sequence-specific manner^19,20^. We hypothesized that more extensive remodeling of the TRS network may better disrupt the finely tuned network of abundant and low-frequency transcripts, altering the natural regulation of global gene expression, and thereby leading to decreased virulence. In addition, it was possible that the virulence observed in CRG3 P6 isolates was due, at least in part, to mouse adaptation during passage. Therefore, to evaluate the effects of more extensive TRN rewiring in a lethal pathogenic model and to further stabilize the TRN against recombination repair and mutation selection, a TRN mutant was constructed that featured the following: (1) the set of 6 mouse-adapted mutations present in the SARS-MA15 virus (see Fig. 4a); and (2) a newly designed TRN consisting of a 7-nt complete replacement of the 9 SARS-CoV TRS loci (ACGAAC to UGGUCGC – CRG7-MA – Fig. 1). These replacements yielded infectious CRG7-MA virus that was capable of replicating to titers equivalent to those of the mouse-adapted WT background virus, SARS-MA15, though with a pinpoint plaque phenotype that remained stable after tissue culture passage. CRG3-MA (9 CRG3 replacements in the virulent SARS-MA15 backbone) was also generated as a control TRN-rewired virus. northern blot analysis of CRG7-MA versus CRG3 and SARS-MA15 revealed that the virus produced the expected bands at similar proportions to WT virus, although some additional low-abundance transcripts were also noted in CRG7-MA (Supplementary Fig. 4).

**Fig. 5:**
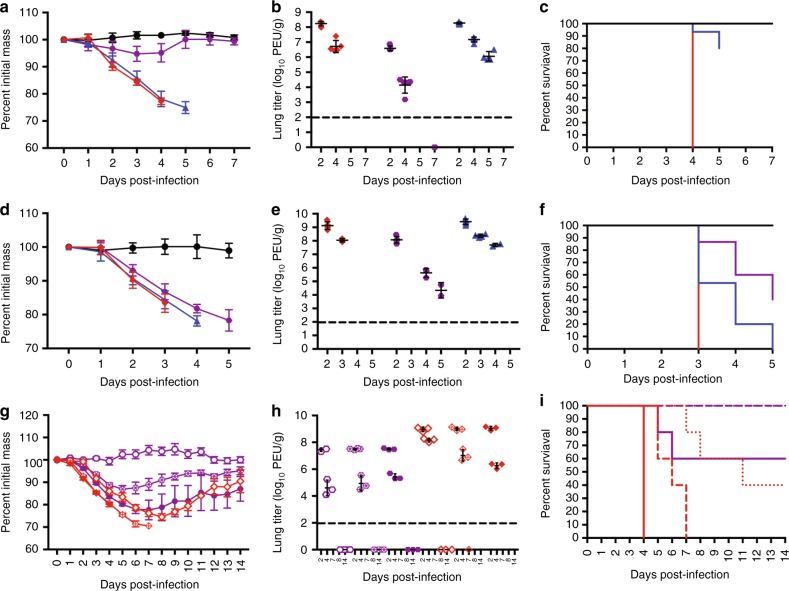
In vivo characterization of the SARS-CoV CRG7-MA mutant. **a**, **d** Weight loss is depicted as percent initial mass in (**a**) young and (**d**) aged BALB/c mice. **b**, **e** Viral replication in the lungs is depicted as titer (log_10_ PFU/g) in (**b**) young and (**e**) aged BALB/c mice. Black circles: PBS; red diamonds: SARS-MA; blue triangles: CRG3-MA; purple circles: CRG7-MA. **c**, **f** Mortality is depicted as percent survival in (**c**) young and (**f**) aged mice. Black: PBS; red: SARS-MA; blue: CRG3-MA; purple: CRG7-MA. **g**–**i** Dose-dependent pathogenicity of SARS-CoV CRG7-MA. Aged BALB/c mice were infected with SARS-CoV CRG7-MA and were evaluated for (**g**) weight loss, depicted as percent initial mass, (**h**) viral replication in the lungs, depicted as titer (log_10_ PFU/g), and (**i**) mortality, depicted as percent survival. Red: SARS-MA; purple: CRG7-MA; unfilled symbols: 2-log_10_ infection; starred symbols: 3-log_10_ infection; solid symbols: 4-log_10_ infection. Dashed lines: limits of detection. Error bars depict standard deviation (**a**, **b**, **d**, **e**, **h**) or standard error of the mean (**g**)

These viruses were then evaluated for replication and virulence in young (10-week-old) and aged (12-month-old) BALB/c mice in comparison with SARS-MA15. In young mice, CRG7-MA was attenuated compared with CRG3-MA and SARS-MA15, causing a weight loss of ~5% (*P* *<* 0.0001, *t* test, on days 2–5 p.i. for CRG7-MA vs. CRG3-MA), though replicating to high titers (approximately 7 log_10_ PFU for CRG7-MA vs. 8 log_10_ PFU for CRG3-MA and SARS-MA15) at 2 days p.i. Further, CRG7-MA showed evidence of more rapid clearance kinetics, with a nearly 3-log_10_ difference in titer compared with the CRG3-MA and SARS-MA15 controls at 4 days p.i. (Fig. 5a–c) (*P* < 0.001, *t* test, for both 2 and 4 days p.i. for CRG3-MA vs CRG7-MA). While morbidity and mortality were more pronounced in old mice, CRG7-MA was still attenuated compared with CRG3-MA and SARS-MA15 in terms of weight loss (less weight loss on days 2–4 p.i., *P* < 0.05 on days 2 and 4 p.i., *t* test, CRG3-MA vs. CRG7-mA) and lung titer (8 log_10_ PFU for CRG7-MA vs. >9 log_10_ PFU for CRG3-MA and SARS-MA15 on day 2 p.i. (*P* < 0.0001, *t* test, CRG3-MA vs. CRG7-MA); ~2.5-log_10_ PFU difference in titer on day 4 p.i. (*P* < 0.05, *t* test, CRG3-MA vs. CRG7-MA) (Fig. 5d–f). Infection of aged BALB/c mice with 2 log_10_, 3 log_10_, or 4 log_10_ PFU of CRG7-MA or SARS-MA15 emphasized the attenuated virulence phenotype, with the 2-log_10_ PFU infection of CRG7-MA causing almost no weight loss over the course of the infection, despite replicating to the same titers as the 3 log_10_ and 4 log_10_ PFU infections (Fig. 5g–h). Under identical conditions, SARS-MA15 mice lost more body weight (>20%) and experienced 60% mortality rates. Survival was markedly different between CRG7-MA and SARS-MA15 infections, with 100% of mice infected with 2 log_10_ or 3 log_10_ PFU and 60% of mice infected with 4 log_10_ PFU surviving the course of CRG7-MA infection. In contrast, only 40% of mice infected with 2 log_10_ PFU of SARS-MA15 survived infection; mice infected with 3 log_10_ or 4 log_10_ PFU survived only until days 6 or 3 p.i. maximum, respectively (*P* < 0.0001, log-rank test) (Fig. 5i). When CRG7-MA was serially passaged in young (10-week-old) BALB/c mice, in contrast to the rapid phenotypic reversion observed with CRG3, CRG7-MA remained phenotypically stable with passage, with mice losing a minimal (~5%) amount of their starting weight over the 3-day infections throughout all 4 passages (Supplementary Fig. 5A). Viral titer also remained stable over passage, with >5 log_10_ PFU detectable at 3 days p.i. in each passage (Supplementary Fig. 5B). Furthermore, when CRG7-MA was serially passaged independently three times for six passages in aged mice, replicating the passage conditions in which CRG3 reverted to virulence, CRG7-MA did not exhibit an increase in virulence, causing <20% weight loss in infected animals and ~60% mortality upon infection of 12-month-old BALB/c mice with 10^5^ PFU of CRG7-MA post-passage, essentially replicating the mortality shown in aged mice infected with non-passaged CRG7-MA virus and distinct from the 100% mortality caused by wild-type SARS-MA15 infection of aged mice (see Fig. 5i, Supplementary Fig. 5C-D, and Supplementary Table 1).

**Fig. 6:**
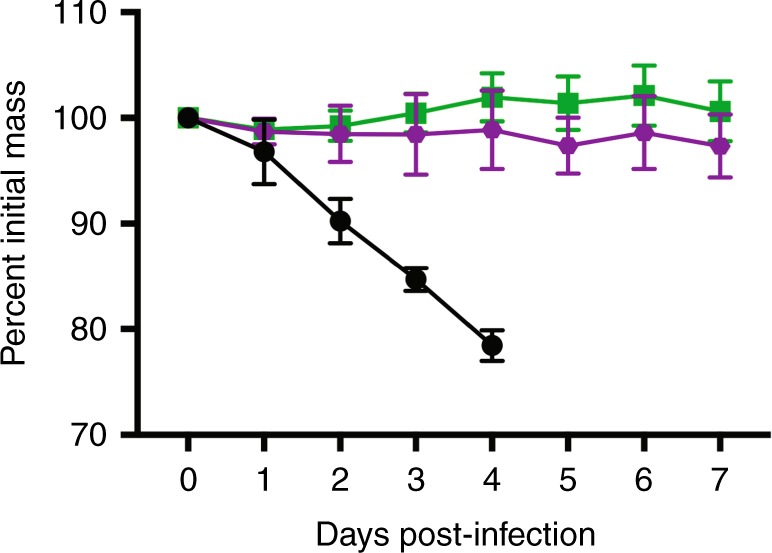
Vaccination of aged mice with SARS-CoV CRG7-MA. Weight loss (depicted as percent initial mass) is shown for aged BALB/ mice vaccinated with PBS, ExoN-MA (control attenuated virus known to protect in an aged model) and CRG7-MA and then challenged with a lethal dose of mouse-adapted SARS-CoV. Vaccinations: PBS, black; ExoN-MA, green; CRG7-MA, purple. Error bars depict standard deviation

Finally, CRG7-MA was evaluated for its capacity to protect against lethal SARS-MA15 challenge in aged (12-month-old) mice. Mice were vaccinated with 2.5 log_10_ PFU of either CRG7-MA or ExoN-MA, which we previously demonstrated to be an effective vaccine in the SARS-MA15 backbone in the aged BALB/c mouse model of SARS-CoV pathogenesis^1^. On day 22 post-vaccination, mice were then challenged with a lethal dose (5 log_10_ PFU) of SARS-MA15 and evaluated for weight loss. Mice lost only minimal amounts (~5%) of their starting weights with both vaccines (*P* = 0.125 for both viruses versus PBS vaccination, Wilcoxon test), indicating that CRG7-MA and ExoN-MA were equally protective against lethal SARS-MA15 challenge (Fig. 6).

## Discussion

Live-attenuated vaccines remain key players in reducing the global disease burden associated with viral infections in humans, critically important livestock, and companion animals. Historically and contemporarily, live-attenuated vaccines have been used with success to help control measles, mumps, rubella, polio, yellow fever, and chickenpox infections and outbreaks^1,21,22^. However, live-attenuated vaccines are also associated with the risk of reversion by either mutation- or recombination-driven processes, which can cause dangerous outbreaks in unvaccinated populations, including animals^22^. For example, highly pathogenic porcine epidemic diarrhea virus (PEDV) strains emerged in China in 2012, circumventing existing vaccines, and RNA recombination events between wild-type and live-attenuated PEDV and between avian infectious bronchitis virus (IBV) strains have seeded new outbreaks^23–26^. Therefore, measures are needed to stabilize live-attenuated vaccines against reversion under selective pressure, particularly for viruses like CoVs, which employ recombination as a standard feature of their replication cycle, as incidental recombination events in the context of a co-infection could unintentionally introduce alleles with enhanced virulence into an attenuated vaccine genome, with consequences that could be difficult to predict^13,27,28^. Several groups, including our own, have developed novel strategies in fidelity regulation and control that attenuate RNA virus pathogenesis, and in the case of CoVs, the development of mutants that prevent reversion repair to virulence^1,29^.

Recombination repair is a well-characterized process essential for genome evolution in many biological systems and plays critical roles in spread, virulence, and pathogenesis^30^. In general, strategies to engineer recombination-resistant RNA viruses have been limited to CoVs and, to a lesser extent, enteroviruses^13,31^. In the context of a CoV infection, recombination occurs when the viral RNA-dependent RNA polymerase (RdRp) switches templates during nascent RNA synthesis, using the nascent RNA itself as a primer once the RdRp reassociates with the template genome. The likelihood of a recombination event occurring is regulated by several viral factors, including replication rates, RNA secondary structure, genome size, and the nature of the replicase/transcriptase protein complex; however, the heritability of a recombination, once it has occurred, is mediated by the replication fitness of the resulting progeny genome^13,32–35^. Several reports have described the emergence of CoVs with enhanced virulence that, upon genome analysis, clearly originated from recombination events of related viruses in avian and mammalian hosts, including SARS-CoV^27,28,36–38^. Furthermore, reservoir species, such as bats, from which the prevailing evidence suggests that both potentially lethal human CoVs, SARS-CoV and MERS-CoV, emerged, have been shown to harbor multiple CoV species, and recombinant RNA genomes have been frequently identified within colonies and among individual infected animals, increasing the likelihood of recombination-driven alterations in species specificity and virulence^16,27,39,40^.

Thus, engineering elements to render a genome recombination-refractory is an essential step towards ensuring that a live-attenuated vaccine candidate cannot regain virulence during an incidental co-infection with another TRN-compatible CoV genome. SARS-CoV is a highly pathogenic pneumo-enteric pathogen that captures many disease features seen among other CoVs. The TRN conserved sequence (CS) motifs utilized in the CRG3 and CRG7 backgrounds, CCGGAU and UGGUCGC, respectively, are both unique sequence motifs when compared with all known CoV genome TRN sequences, greatly reducing and likely eliminating the possibility of recombination with unmodified genomes at the canonical TRS loci. Our previous work demonstrated that introducing mismatched TRSs was lethal for RNA recombinant virus replication^13^. Furthermore, here, we showed that CRG3 TRN replacement did not revert at the primary sites of mutation, indicating that the rewired TRN is stable and not under sufficient selective pressure to revert, even in vivo. As coordinated interactions are required for TRN function, TRN reversion is unlikely, given the requirement for nearly simultaneous reversions at multiple sites across the genome. Larger-sized TRS CSs (>7 nts) within the TRN may not prove effective, as evolution appears to have selected for a CoV RNA polymerase that is heavily focused on recognizing a 5- to 7-nt TRS CS within the TRN to regulate subgenomic transcription.

The attenuation resulting from TRN rewiring is most likely attributed to alterations in the viral transcription profile, low-abundance transcripts that either encode or reduce the expression of previously unidentified out-of-frame ORFs, or viral or host factor interactions with the viral genome. The rewired TRN produces obvious novel viral RNA species (Supplementary Fig. 4). These novel RNA species may serve as functional mRNAs, producing noncanonical viral protein products that attenuate viral replication or pathogenesis. Alternatively, these novel RNA species may compete with canonical viral RNA species for replication and transcription, attenuating viral replication and/or pathogenesis due to the altered availability of transcripts encoding bona fide viral virulence factors. Furthermore, the rewired TRN itself may serve as an attenuating factor, as the virus’ discontinuous transcription program alters programmed RNA-protein interactions (involving both viral and host proteins) either directly (i.e., by altering the bases required for RNA-protein interactions) or indirectly (i.e., through changes in the RNA’s secondary structure that affect the steric availability of RNA-protein interaction sites). Moreover, Di et al., in an arterivirus model, used next-generation sequencing analysis to show that noncanonical transcripts are produced in the course of wild-type infection, indicating that the coding capacity of nidoviruses is actually much larger than what has been characterized using Sanger sequencing and biochemical detection methods. Their findings suggest that attenuation via the TRN may be able to target noncanonical RNA species, which might be able to impact pathogenesis with fewer effects on replication and structural protein production^41^. These fascinating possibilities will be the focus of future studies on the mechanism of TRN-related attenuation.

The hypothesis that alterations in the expression profiles of canonical viral RNA species and protein products attenuate pathogenesis is further strengthened when paired with observations of the types of mutations that were selected upon passage of CRG3 in aged mice: mutation profiles were different if selection occurred in young versus aged populations. Frieman et al. previously demonstrated that mutations in Spike and nonstructural protein 9 (nsp9) were repetitively selected in several independent passages and that these mutations conferred virulence in young mice, suggesting that Spike–viral receptor interactions and nsp9–replicase protein interactions were most important for virulence^42^. In severely ill SARS-CoV-infected humans (predominantly over 50 years of age), deletions in and around ORF8 were identified, as discussed earlier^16–18,39^. Deletions of different accessory ORFs were the most frequently observed changes after selection in aged animals. Furthermore, in aged mice, nearly any combination of 2 SARS-MA15-identified alleles (shown in Fig. 4a) also conferred increased virulence, most likely reflecting the increased susceptibility of aged mice to lethal outcomes. These data suggest that CoV adaptation to virulence is different in young and aged animals, especially when coupled with variations in virus pathogenic determinants. While immune senescence can enhance virus virulence^43^, our data support the novel hypothesis that virulent zoonotic coronaviruses may emerge more quickly after in vivo passage, especially in the aged, where multiple evolutionary pathways exist that can program virus virulence.

The disproportionate identification of mutations in the accessory ORFs following passage of the CRG3 TRN virus in aged animals may reflect the roles these accessory ORFs are hypothesized to play in the modulation of host immunity^44^. Aged animals’ immune systems respond differently, and usually more severely, to microbial challenge, with the immune response skewed towards severe innate immune effects and defects in adaptive immunity^45^. However, in the context of CoV infection, this augmented immune response, when presented in the context of accessory ORF deletion, may serve as a virulence fulcrum, with the balance poised between essential immune recognition and severe disease, possibly due to defects in spread and persistence. These potential effects on host immunity argue that a stable vaccine candidate should consider the potential for changes in accessory ORFs.

A well-designed vaccine candidate should include genetic traps that are either independently attenuating or triggered by recombination events. We have previously demonstrated both stable attenuation and protection against lethal challenge with inactivations of the nsp14 exonuclease^1^ and the nsp16 2′-O-methyltransferase^46^ activities. In addition, Züst et al. demonstrated that a partial deletion of the murine hepatitis virus (MHV) nsp1 replicase protein could protect against homologous and heterologous challenge^47^. Combining alleles that render the virus recombination-refractory, alter RNA replication fidelity, and that result in an altered host immune response could produce stable, reversion-proof, live-attenuated viruses that induce robust neutralizing immunity. Attenuating alleles, coupled with a rewired TRN, are anticipated to increase the stability of the attenuation and to minimize the chances for RNA recombination repair. The strategies reported herein, coupled with the availability of new molecular clones for CoVs that cause severe disease in livestock populations, provide a vehicle for improved live virus vaccine design^48–53^. It may also be possible to “tune” the CoV TRN, with attenuating changes concentrated on virulence alleles, such as accessory genes known to impact pathogenesis. Such alterations have the potential to greater enhance the stability of a vaccine candidate, particularly if considering TRS context outside of the 5- to 7-nt core sequence. Such studies would benefit the most if the RNA structure is also considered, as TRS accessibility is most likely modulated by structural elements in the CoV RNA genome. Such studies will be the focus of future work.

With the continuing identification of zoonotic pools of CoVs that genetically resemble lethal human and animal CoVs, often with only a few percentage points of difference between the zoonotic and lethal human sequences^10,54–57^, the necessity for a rapidly implementable, universal attenuation platform for CoV live-attenuated vaccine design is underscored. In this report, we described the design and implementation of a CoV attenuation strategy that can be easily and rapidly adapted to any CoV genome. The presence of 8–9 characterized TRSs within any CoV genome, the CSs of which are 6–7 nts each, offers too large and complex a target for primary site reversion to be a likely event. As most single recombination events would decouple TRN expression networks, these recombinants would be lethal. Therefore, this attenuation strategy, when paired with alleles that can resist selection events that lead to second-site reversion, could bring live-attenuated CoV vaccines within the reach of realization in the face of the ever-growing threat of new human and animal CoV-based epidemics.

## Methods

### Viruses and cells

All virus stocks were propagated in Vero-E6 cells as described in^58^. All virus work was performed in a biological safety cabinet in a biosafety level 3 laboratory.

### Construction of SARS plasmids and viruses

TRN3-based plasmids were constructed in^13^. TRN7 mutations were introduced into SARS plasmids A and F using cassettes generated by BioBasic. To generate SARS-F plasmids containing both TRN mutations and the mouse-adapted mutation at nt 2663, the mouse-adapted mutation was cloned into the F TRN plasmids via a PCR and restriction digestion strategy. To generate SARS-F plasmids containing both TRN mutations and the mouse-adapted mutation at nt 2663, the mouse-adapted mutation was cloned into the F TRN plasmids via a PCR and restriction digestion strategy. The plasmids F-BstZ (5′-GGAGGCGCAATTTTTGTACCTCTATGCCTTG-3′), R-MAmut (5′-AGCTATCGTCTCCGCTTCTCAACGGTAATAGTACCGTTGTCTG-3′), F-MAmut (5′-AGCTATCGTCTCCAAGCTTAAACAACTCCTGGAACAATGGAAC-3′), and R-Msc (5′-GTGGCTTAGCTACTTCGTTGCTTCCTTCAGGC-3′) were used to generate 2 amplicons. The resulting amplicons were restriction-digested with *Bsm*B I, ligated, and purified, after which the ligated amplicons and the parent vectors were restriction-digested with *BstZ17* I and *Msc* I and ligated. Ligated vectors were transformed into TopTen *E*. *coli* cells, and the resulting colonies were screened and sequence-verified. Viruses were then constructed as described in ref. ^58^.

### Northern blot analysis

Intracellular RNA was isolated using RiboPure reagents (Ambion, Austin, TX) 12 h post-infection^13^. The mRNA was then isolated using a Qiagen Oligotex mRNA isolation kit, treated with glyoxal, and separated an agarose gel using NorthernMax-Gly (Ambion). The RNA was then transferred to BrightStar-Plus membrane (Ambion) for 5 h, cross-linked using UV light, prehybridized, and probed with an N gene-specific oligonucleotide probe (5′-CTTGACTGCCGCCTCTGCT^b^T^b^CCCT^b^CT^b^GC^b^-3′; biotinylated nucleotides are denoted with a superscripted “b”). The blot was hybridized overnight and washed with low- and high-stringency buffers and was then incubated with phosphatase-conjugated streptavidin. The blot was then incubated with CDP-STAR, overlaid with film, and developed.

### Mouse infections with SARS-CoV and mutants

All experimental protocols involving mice were reviewed and approved by the institutional animal care and use committee at the University of North Carolina, Chapel Hill, NC, USA. The following mice were used: 10-week-old female BALB/c (Charles River Laboratories, Wilmington, MA, USA) and 14-month-old female BALB/c (Harlan Laboratories, Indianapolis, IN, USA). Mice were lightly anesthetized and infected intranasally with varying doses (10^2^–10^6^ PFU, depending on the experiment) of SARS-CoV, MA15, or TRN mutants. Mice were weighed daily, and on certain days specified in each experiment, mouse lungs were harvested for virus titer and/or RNA. Serial passages were inoculated as above for passage 1; subsequent passages were inoculated with 50 μL of clarified lung homogenate (lungs were homogenized in 1 mL of PBS) from the previous passage. All experiments used a minimum of *n* = 5 mice per virus per dosage/condition (if applicable) per timepoint. For infection–challenge studies, mice were infected with 10^2^–10^3^ PFU of the indicated vaccine virus, weighed for the 7 days following initial infection, and then challenged with a lethal dose (10^6^ PFU) of MA15 for the challenge infection.

### Determination of virus titer in infected mouse lungs

Lungs harvested for virus titer were weighed and homogenized in 1.0 mL of PBS at 6000 rpm for 60 s in a MagnaLyser (Roche, Basel, Switzerland). Virus titers were determined by plaque assay on Vero cells.

### Determination of viral neutralization antibody titers

Mouse sera were heat-inactivated for 30 min at 55 °C and were then serially diluted to 1:100, 1:200, 1:400, 1:800, and 1:1600 in PBS to a volume of 125 μL. Next, 125 μL of PBS containing low-concentration SARS-CoV MA15 (40 PFU) or high-concentration SARS-CoV MA15 (240 PFU) was added to each serum dilution. The virus-serum mixtures were incubated at 37 °C for 30 min. Following incubation, virus titers of the mixtures were determined by plaque assay. Finally, we calculated the 50% plaque reduction neutralization titer (PRNT_50_) values, the serum dilutions at which plaque formation was reduced by 50% relative to that of virus stock not treated with serum.

### Viral genome sequencing

To determine the sequences of viral genomes present in mouse lungs after passage, plaques were isolated from lung samples as described above. Briefly, individual viral plaques were harvested by collecting the agarose plugs above them using a 200-μL pipette tip. Each agarose plug was dropped in 0.5 mL PBS, allowed to diffuse for 24 h at 4 °C, and then applied to ~70% confluent monolayers of Vero-E6 cells in T25 flasks and incubated for 48 h at 37 °C. Infected cell monolayers were then harvested in 1 mL of TRIzol. First-strand cDNA was generated as described in ref. ^59^. Amplicons of the viral genomes were generated as described in ref. ^1^. Sequence results were analyzed using Geneious R11 (Biomatters, Auckland, New Zealand) and Serial Cloner 2.6.1 (SerialBasics, http://serialbasics.free.fr/Home/Home.html).

### Statistical analyses

Statistical analyses were performed using GraphPad Prism 7 (GraphPad Software, La Jolla, CA, USA). The tests run depended on the experimental design and are specified in the text. Significance was set at *P* < 0.05.

## Electronic supplementary material

Supplemental Information

## Data Availability

The viral genome sequence datasets are available via the NCBI GenBank with accession identifiers MK062179 through MK062184. All other data generated during and/or analyzed during the current study are available from the corresponding author on reasonable request.
