# Supplementary material for: Evaluation of a recombination-resistant coronavirus as a broadly applicable, rapidly implementable vaccine platform
Source: Commun Biol. 2018 Oct 29;1:179. doi: 10.1038/s42003-018-0175-7 (PMC6206136; doi:10.1038/s42003-018-0175-7)
Supplement: Supplementary file 1 — Supplemental Information [file 42003_2018_175_MOESM1_ESM.pdf]

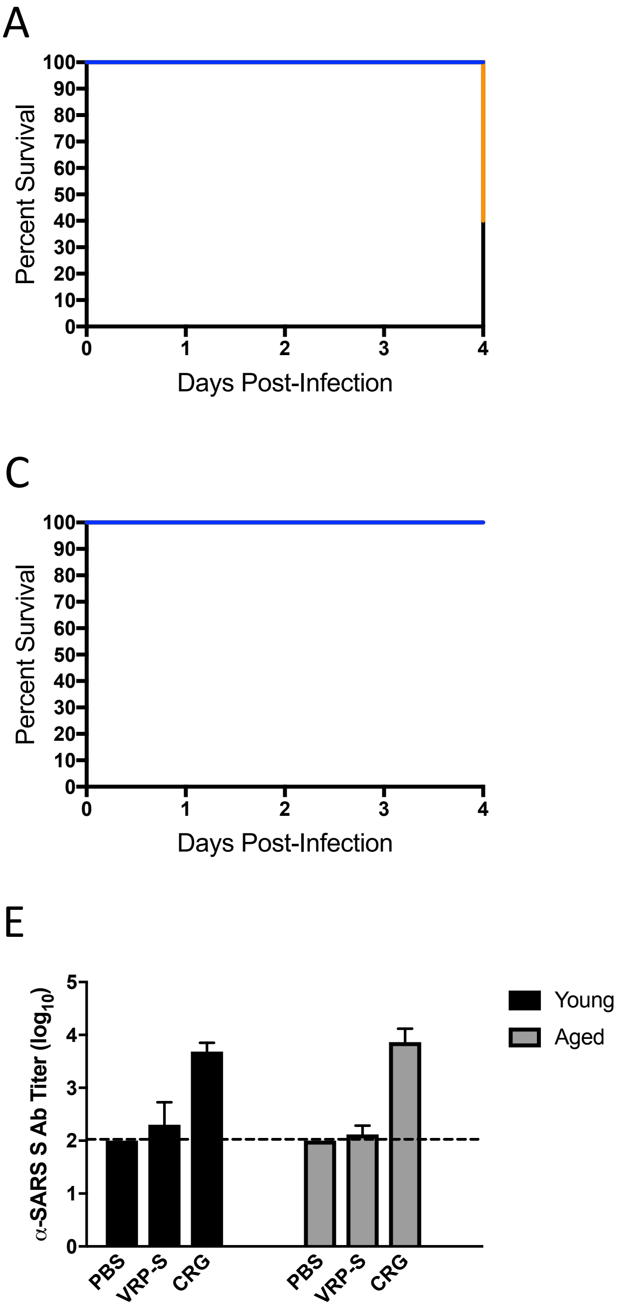

Supplementary Fig. 1. Survival and antibody responses of young and aged mice vaccinated with SARS-CoV CRG3 and subjected to homologous and heterologous challenge. (**A, B, C, D**) Morbidity upon homologous (**A, B**) and heterologous (**C, D**) challenge is depicted as percent survival in (**A, C**) young and (**B, D**) aged vaccinated BALB/c mice. Black: PBS; Blue: CRG3; Orange: VRP-S. (**E**) Neutralizing antibody titers in young (black) and aged (grey) BALB/c mice are depicted as $\log_{10}$ serum antibody titers. Error bars depict standard deviation.

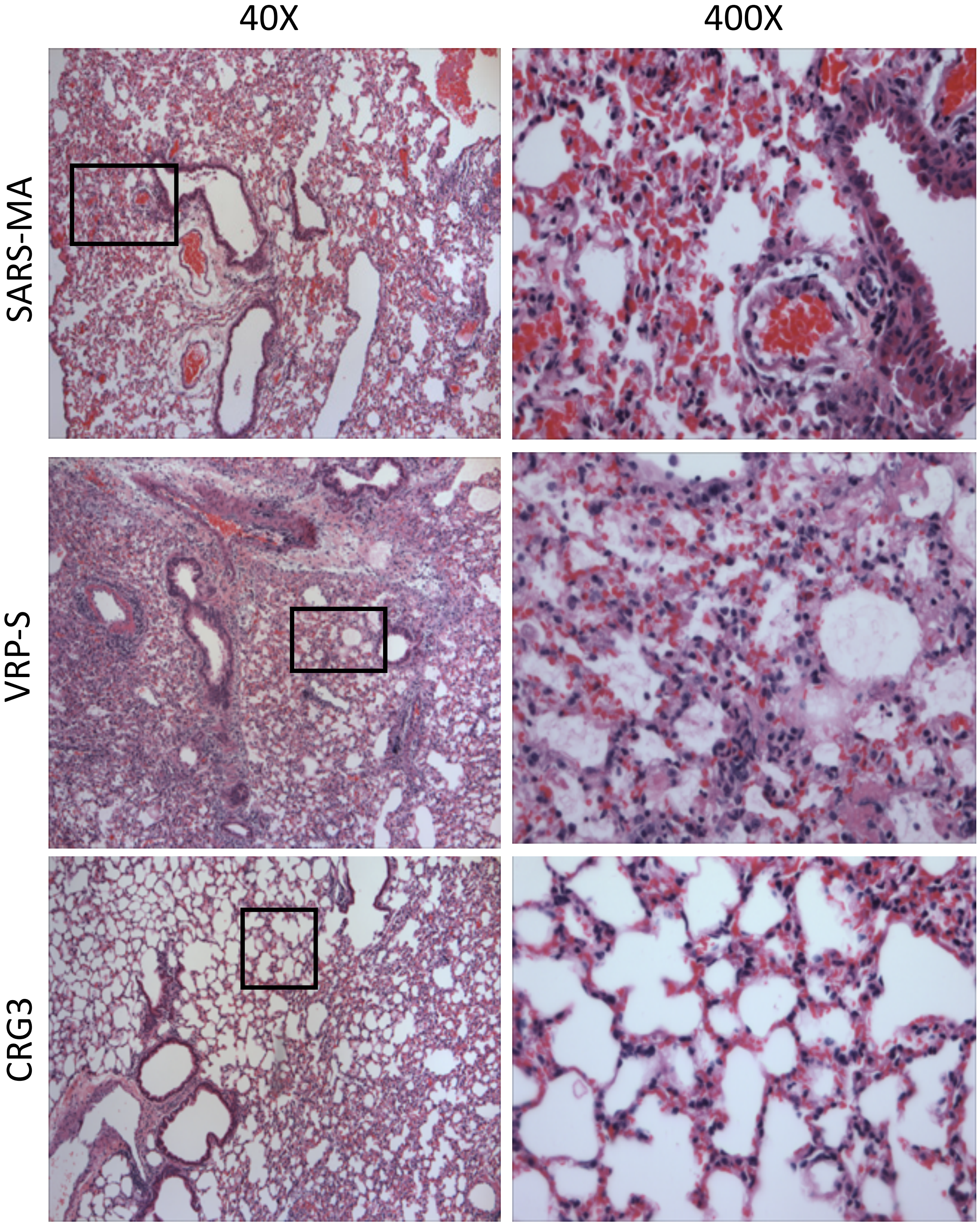

Supplementary Fig. 2. Lung pathology in mice vaccinated with CRG3. Aged mice were vaccinated with a sublethal dose (10^2 PFU) of mouse-adapted SARS-CoV (SARS-MA), viral replicon particles expressing SARS-CoV Spike (VRP-S), or CRG3. Hematoxylin and eosin-stained lung sections from mice (day 7 post-infection) are shown at 40X (left column), with the inset indicated in each image enlarged to 400X (right column).

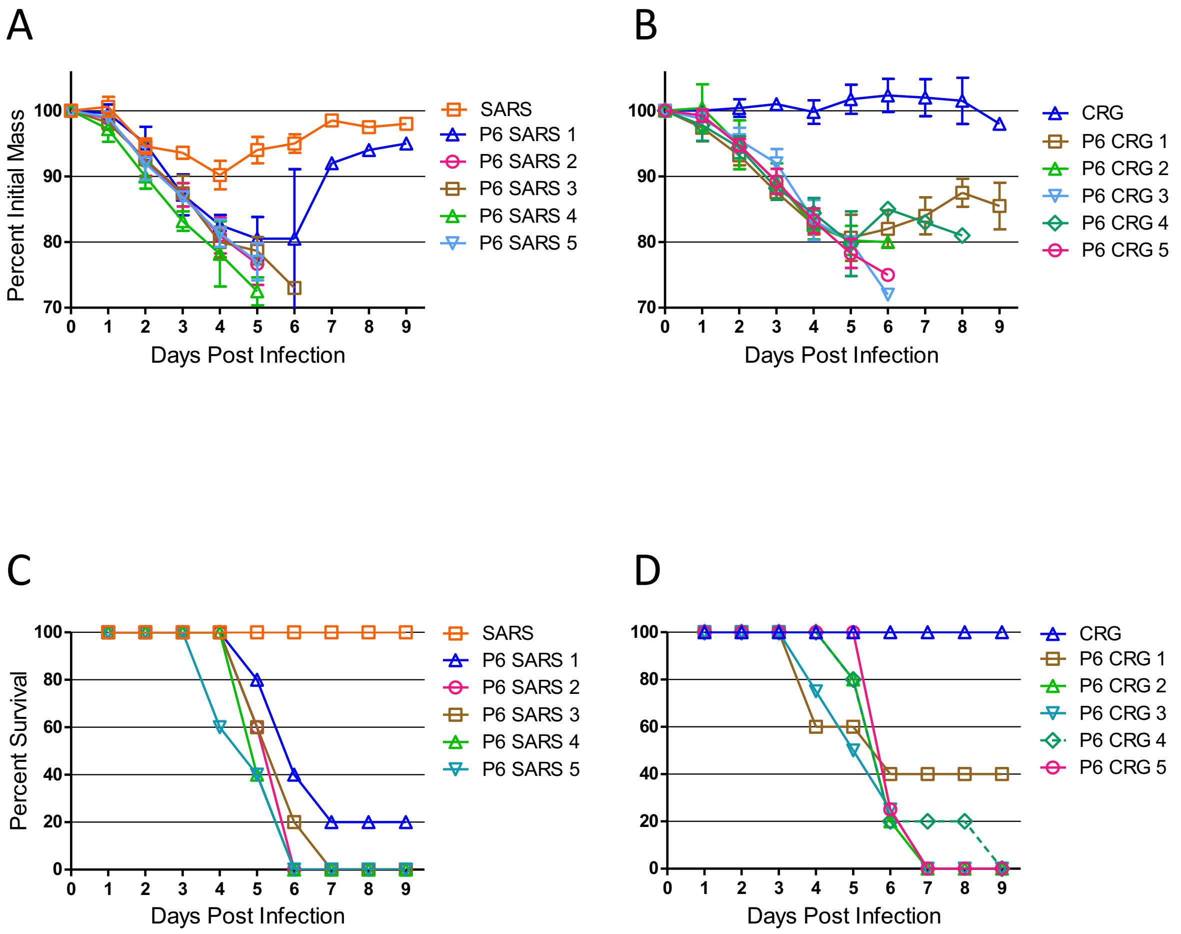

Supplementary Fig. 3. Weight loss and survival in WT and CRG3 following passage. (A, B) Weight loss is depicted as percent initial mass in mice infected with **(A)** WT and **(B)** CRG viruses after 6 passages. **(C, D)** Morbidity is depicted as percent survival in mice infected with **(C)** WT and **(D)** CRG viruses after 6 passages. Error bars depict standard deviation.

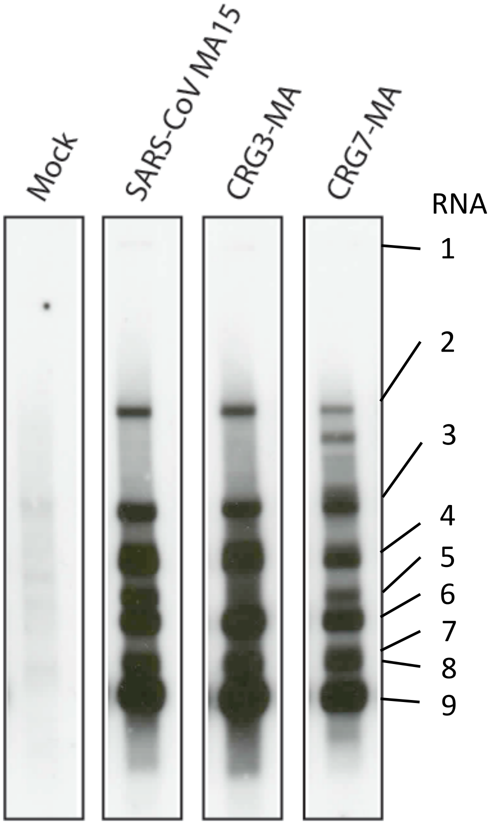

Supplementary Fig. 4. Northern blot analysis of TRN mutants versus wild-type SARS-CoV. Viral RNA was resolved by electrophoresis, transferred to a membrane, and probed with a SARS-CoV N-specific biotinylated probe. RNAs that bound probe were visualized using phosphatase-conjugated streptavidin. Canonical RNA species are numbered (1-9). Note: RNA 1 transfers poorly in Northern blots due to its size.

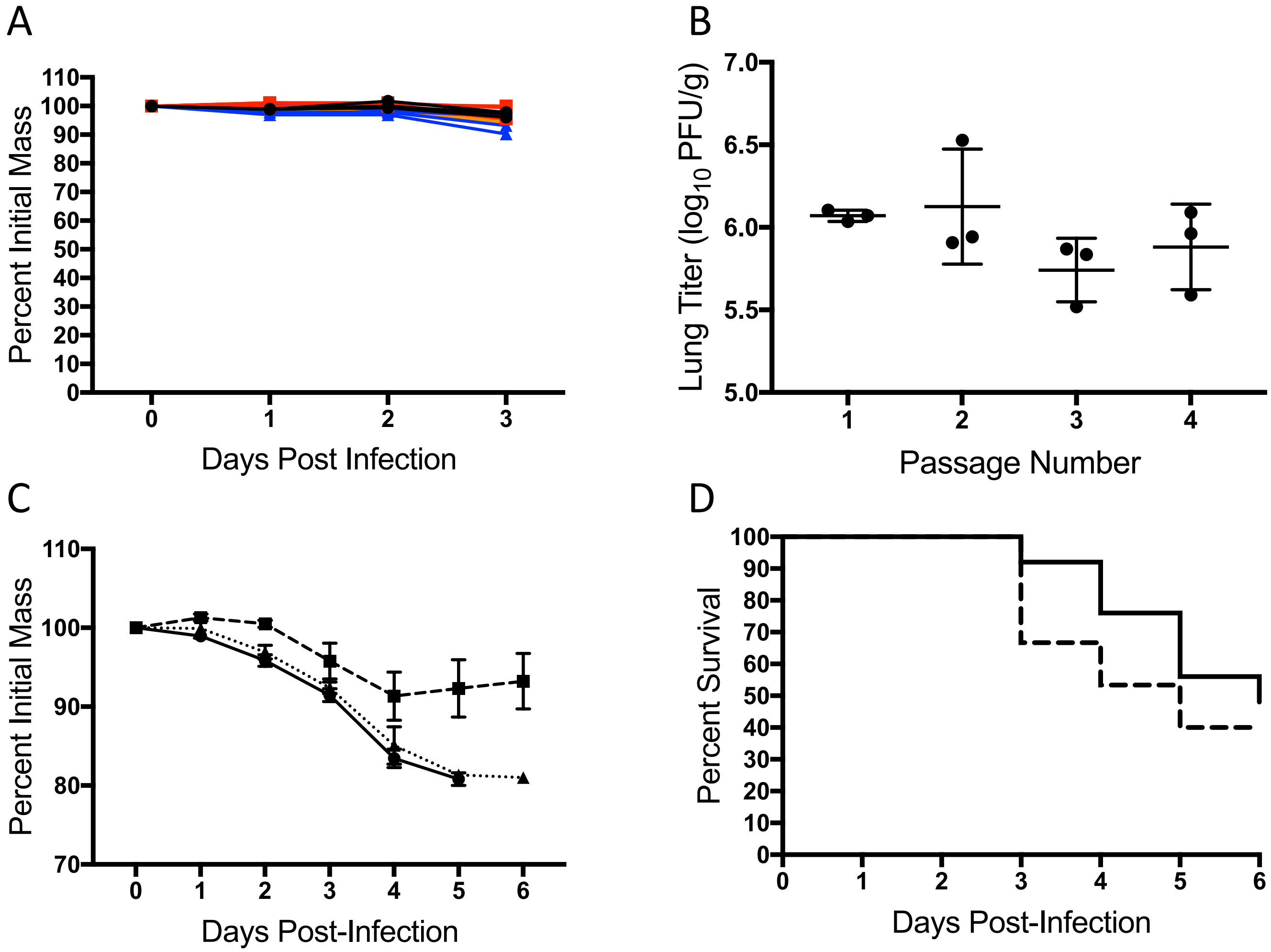

Supplementary Fig. 5. *In vivo* stability of the SARS-CoV CRG7-MA mutant. (**A, B**) After 4 passages of CRG7-MA in young BALB/c mice, the resulting viruses from each passage were evaluated for replication and pathogenesis *in vivo*. (**A**) Weight loss after infection of mice with viruses from each passage is depicted as percent initial mass. Black: Passage 1; red: Passage 2; blue: Passage 3; orange: Passage 4. **(B)** Viral replication in the lungs after infection of mice with viruses from each passage is depicted as titer (log_{10} PFU/g). (**C, D**) After 6 passages of CRG7-MA in aged BALB/c mice, the resulting viruses from each passage were evaluated for pathogenesis *in vivo*. (**C**) Weight loss after infection of mice with three different serially passaged viruses from passage 6 is depicted as percent initial mass. Solid lines: Virus 1; dashed lines: Virus 2; dotted lines: Virus 3. (**D**) Mortality of aged mice infected with CRG7 pre- and post-passage is depicted as percent survival. Solid line: pre-passage; dashed line: post-passage. Error bars depict standard deviation.

	Mortality (%)	
	Young Mice	Aged Mice
WT SARS-CoV	0	0
WT SARS-CoV P6	n.d.	100
CRG3	0	0
CRG3 P6	n.d.	100
SARS-MA	100	100
CRG3-MA	20	100
CRG7-MA	0	52
CRG7-MA P6	n.d.	60

Supplementary Table 1. Summary of SARS vs. CRG mortality *in vivo*. Mortality in young and aged mice is indicated for all viruses described in the manuscript as percentages. n.d.: not determined.
